# Maternal DOT1L is dispensable for mouse development

**DOI:** 10.1038/s41598-020-77545-6

**Published:** 2020-11-26

**Authors:** Ji Liao, Piroska E. Szabó

**Affiliations:** Center for Epigenetics, Van Andel Research Institute, Grand Rapids, MI 49503 USA

**Keywords:** Developmental biology, Genetics

## Abstract

A battery of chromatin modifying enzymes play essential roles in remodeling the epigenome in the zygote and cleavage stage embryos, when the maternal genome is the sole contributor. Here we identify an exemption. DOT1L methylates lysine 79 in the globular domain of histone H3 (H3K79). *Dot1l* is an essential gene, as homozygous null mutant mouse embryos exhibit multiple developmental abnormalities and die before 11.5 days of gestation. To test if maternally deposited DOT1L is required for embryo development, we carried out a conditional *Dot1l* knockout in growing oocytes using the *Zona pellucida 3-Cre (Zp3-Cre)* transgenic mice. We found that the resulting maternal mutant *Dot1l*^*mat−/*+^ offspring displayed normal development and fertility, suggesting that the expression of the paternally inherited copy of *Dot1l* in the embryo is sufficient to support development. In addition, *Dot1l* maternal deletion did not affect the parental allele-specific expression of imprinted genes, indicating that DOT1L is not needed for imprint establishment in the oocyte or imprint protection in the zygote. In summary, uniquely and as opposed to other histone methyltransferases and histone marks, maternal DOT1L deposition and H3K79 methylation in the zygote and in the preimplantation stage embryo is dispensable for mouse development.

## Introduction

The earliest stages of embryo development entail the reprograming of the epigenome in the zygote from the gamete-specific programs of the oocyte and the sperm to totipotency, then zygotic genome activation, and cleavage^1–3^. Early developmental processes predominantly depend on the supplies provided into the oocyte by the maternal genetic component. Such maternal effects are detected genetically when inactivation of specific genes in the oocyte results in a developmental phenotype^4^. Because enzymes that catalyze the formation, or removal of covalent modifications of DNA and histone molecules carry out epigenome reprogramming in the early embryo, maternal effect of phenotype is observed for genes that encode chromatin-modifying enzymes^5–15^.

Disruptor of telomeric silencing 1 (*Dot1*) was identified in a yeast genetic screen for suppressors of telomeric silencing^16^. Overexpression, as well as inactivation of *Dot1* suppressed silencing in position effect variegation (PEV) assays using reporter transgenes integrated at specific telomeric and pericentromeric loci^15,17–19^. Later it was shown using H3K79 methylation-defective mutants and genome-wide expression analysis, that telomeric and pericentromeric silencing is not generally affected by *Dot1*, as most telomeric and centromeric genes are not subject to H3K79 methylation-dependent natural silencing^19^. *Dot1* catalyzes mono-, di-, and tri-methylation of lysine 79 (H3K79), a residue that is exposed on the surface of the nucleosome in the globular domain of histone 3^16–19^. The H3K79 methyltransferase function is conserved in the orthologous proteins, GRAPPA and Dot1-like (*DOT1L*) in the fruit fly and mouse, respectively^20–22^. Loss of function of *Dot1l* and *gpp* lead to complete loss of H3K79 methylation, revealing that DOT1L is the sole enzyme in the mouse that methylates H3K79^20,22^. *Dot1/DOT1L* plays roles in transcriptional regulation, cell cycle control, and DNA damage response^18,20,23–25^. The enzymatic steps of DOT1 in generating the H3K79me1, H3K79me2, and H3K79me3 states, are genetically controlled, at least in the yeast^18^. H3K79me2/me3 is considered an active chromatin mark based on ChIP-chip analysis^26^. Mixed lineage leukemia gene (MLL) rearrangements (MLL-r) are a major cause of incurable acute lymphoblastic leukemias (ALL). Using a DOT1L inhibitor in MLL-AF4 leukemia cells, H3K79me2/3 was found to be required for maintaining chromatin accessibility, histone acetylation and transcription factor binding, and also for maintaining enhancer-promoter interactions at a subset of H3K79me2/me3 enriched enhancers^27^. Several lines of evidence suggest, however, that the two marks, H3K79me2 and H3K79me3, may have the opposite readout with regards to gene regulation. Interestingly, *gpp* mutant flies not only exhibit phenoytypes that are consistent with defects in polycomb-type gene repression, but also those with trithorax-type gene activation. These contradictory phenotypes may result from loss of *gpp* activity in mutants at sites of both active and inactive chromatin domains^21^. Indeed, whereas H3K79me2 is found in puffs and interbands of *Drosophila melanogaster* salivary gland polytene chromosomes, where gene activity is observed, H3K79me3 is mainly located in the bands that are inactive. In mouse somatic cells and oocytes, H3K79me2 staining was detected throughout the genome by immunocytochemistry. Whereas H3K79me3 was localized in the pericentromeric heterochromatin regions, which are devoid of active genes^28^. We showed by immunostaining that the global levels of globular histone marks H3K79me3 and H3K79me2 strongly increased in male but not female mouse fetal germ cells at 15.5 dpc, at the time when epigenome remodeling takes place in the male germ cells^29^. We also found that H3K79me3 is localized to DAPI-stained heterochromatin whereas H3K79me2 is localized to DAPI-poor regions in mouse germ cells and somatic cells of the fetal gonad^29^ In addition, we found that H3K79me3 and H3K79me2 generally cluster with repressive and active histone marks, respectively in chromosome-wide ChIP-chip mapping in mouse fibroblasts^30^.

Genomic imprinting allows one parental copy of a gene to be expressed while the second allele is silenced, providing a useful paradigm for understanding epigenetic regulation. We found that H3K79me3 and H3K79me2 occupy reciprocal methylated versus unmethylated parental alleles of differentially methylated regions (DMRs) in the mouse^31^. Maternal genomic imprints at DMRs are established in the growing oocytes^32,33^. Based on earlier genetic studies^34–38^ we expect that maternal mutation of *Dot1l* in the growing oocytes would eliminate the imprinted expression at those genes where the establishment of maternal imprints or the maintenance of maternal or paternal imprints depended on DOT1L.

Whereas *Dot1* is not an essential gene in the asexual vegetative life cycle of *Saccharomyces cerevisiae*^17^, genetic mutations revealed that the orthologous genes are essential in the soma of multicellular organisms such as *Drosophila melanogaster*, and *Caenorhabditis elegans*^21,39^. DOT1L is ubiquitously present in the mouse embryo, suggesting its important function in mouse embryonic development. Deletion of the methyltransferase catalytic domain of DOT1L (exons 5 and 6) by gene targeting resulted in embryonic lethality between 9.5 and 10.5 days post coitum (dpc) due to development abnormalities including stunted growth, defective yolk sac angiogenesis, and dilation of the heart^22^. Another *Dot1l* knockout model, disrupted the nucleosome binding domain (exon 13) and was embryonic lethal between 10.5 to 13.5 dpc, due to defects in early hematopoiesis. Vessel-remodeling defects were also observed in the extraembryonic tissues, which appeared as the consequence of altered hematopoiesis and reduced blood flow in these embryos^20^. In addition to the embryonic phenotypes, tissue specific inactivation of *Dot1l* revealed that DOT1L is important in several fetal and adult organ systems including erythropoiesis, brain development, and cartilage development^24,40–43^. DOT1L pays a role in the development of the cortical plate, by maintaining the progenitor pool, and by affecting the cortical distribution of neurons. Mechanistically, DOT1L activity promotes transcription of genes implicated in asymmetric cell division^24^.

It is not known whether DOT1L has a maternal effect of phenotype. Several lines of evidence predict that DOT1L may be important in the very early stages of embryo development, which depend on maternally supplied DOT1L protein stored in the oocytes. *Dot1l* transcription and DOT1L protein are present in mouse oocytes and preimplantation embryos^44^, and *Dot1l* RNA and DOT1L protein levels decrease from the oocyte by the 2-cell stage, remain low at 4-cell stage, and then increase by the blastocyst stage. This pattern suggests that the DOT1L deposition from the oocyte persists during early preimplantation development, and embryonic *Dot1l* gene activity turns on after the four-cell stage. The *Drosophila* ortholog, GRAPPA is present in *gpp* zygotic mutant fly embryos. Interestingly, the severity of the *gpp* developmental phenotype was enhanced in the fly when the mothers of homozygous mutant males were homozygous for the *gpp* mutation compared to those from heterozygous mothers^21^. Ooga et al.^28^ showed that in the mouse, both H3K79me2 and H3K79me3 decrease soon after fertilization, and the hypomethylated state is maintained in the blastomeres at interphase, except for a transient increase in H3K79me2 at mitosis (M phase). However, H3K79me3 is not detectable throughout preimplantation, even at M phase. H3K79me2 is lost during somatic nuclear transfer into activated oocytes^28^. These results suggested that rapidly eliminating H3K79 methylation after fertilization is involved in remodeling the epigenome of the fully differentiated oocyte into the totipotent state, which then gives rise to the embryo soma.

Microinjection experiments revealed that (FLAG-tagged) DOT1L is localized in the nucleus at the one-cell and four-cell stages but not at the two-cell stage. This dynamic pattern involves an active export mechanism of DOT1L from the nucleus in 2-cell embryo that requires the C-terminus of DOT1L^44^. DOT1L relocation may be required to regulate the formation of chromocenters (or pericentromeric heterochromatin clusters). Major satellites are found associated with the nucleolar precursor bodies in the one-to two-cell embryos, but they are dissociated from NPB-s and then reassembled in the nucleoplasm after the four-cell stage. Forced expression of the DOT1L caused H3K79 hypermethylation and the formation of chromocenter-like structures at the two-cell stage, and developmental arrest of the embryos at the two-cell stage. These results suggest that the absence of DOT1L, and perhaps the avoidance of chromocenters at the two-cell stage, is essential for early preimplantation development. Similar to the natural events during preimplantation development, inhibition of DOT1L accelerated reprogramming of somatic cells into induced pluripotent stem cells. Reduced H3K79 methylation correlated with the silencing of lineage-specific genes, as well as the upregulation of certain pluripotency genes, such as NANOG and LIN28. This finding is consistent with a role of DOT1L as a barrier to reprogramming^45^.

The question arises, whether any of these processes is essential for embryo development: (1) broad presence of H3K79me2 in the oocyte and in the maternal pronucleus, (2) the elimination of H3K79me2 shortly after fertilization, (3) localization of H3K79me3 at the pericentric heterochromatin of germinal vesicle oocytes and metaphase I oocytes (4) reassembly of H3K79me2 to the chromosomes at the M-phase during preimplantation, (5) removal of DOT1L from the nucleus in the process of eliminating chromocenters at the 2-cell stage; (6) a role of DOT1L/H3K79me2 in the newly forming chromocenters at the four-cell stage. We expected to answer some of these questions by a maternal *Dot1l* knockout experiment. We inactivated *Dot1l* in the growing oocytes and assessed the developmental potential of the resulting embryos. Surprisingly, we found that maternal contribution of DOT1L in the egg is dispensable from the growing oocyte stage through fertilization and up to the 4-cell stage of mouse embryo development.

## Results

### Mouse model

**Figure 1:**
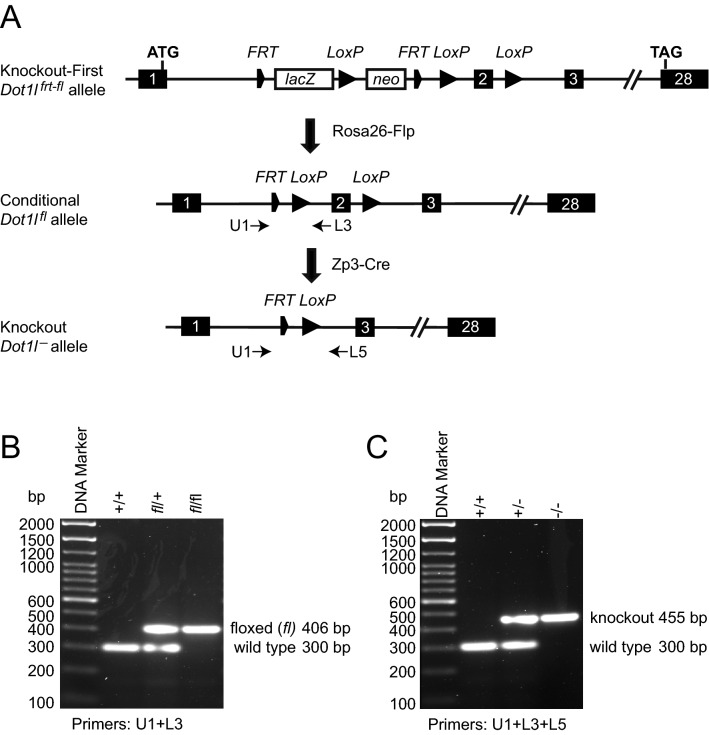
*Dot1l* knockout mouse model. (**A**) The gene targeted Knockout First allele of the *Dot1l*^tm1a(KOMP)Wtsi^ strain from the KOMP Repository is shown. Exons are numbered. The *frt*-flanked LacZ-neo gene-trap cassette was removed by FLPe recombinase, resulting the *Dot1l*^fl^ conditional allele. The floxed second exon of the *Dot1l* gene was then excised by the *Zp3* promoter-driven Cre recombinase in growing oocytes, causing a frame-shift mutation and resulting in a null allele. (**B**) PCR genotyping results are shown to detect the *Dot1l*^fl^ conditional allele in mice. (**C**) PCR genotyping to detect the knockout allele. The primers used in these assays are marked in the maps above. The un-cropped gel image is provided in Figure S1.

**Figure 2:**
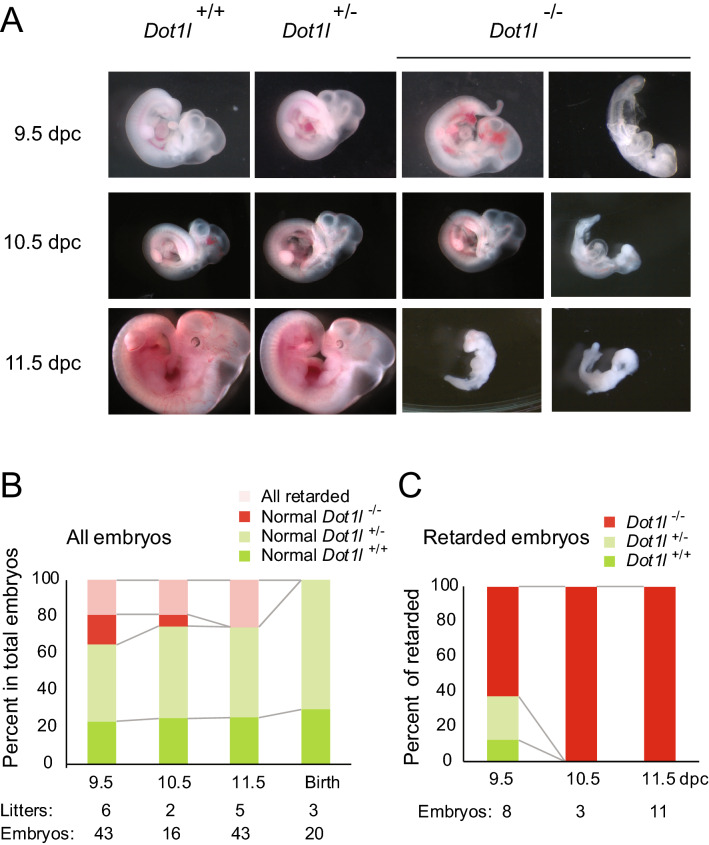
Homozygous *Dot1l*^*−/−*^ mutant embryos die between 9.5 and 10.5 dpc. (**A**) Representative pictures of *Dot1l*^+*/*+^, *Dot1l*^+*/-*^ and *Dot1l*^*−/−*^ embryos from a *Dot1l*^+*/−*^ intercross are shown at different developmental stages, as indicated to the left. (**B**) The chart displays the distribution of the normal embryos according to the three genotypes, and the retarded embryos among all genotypes at each time point. (**C**) The chart displays the percentages of each genotype among the retarded embryos at each time point.

We obtained a targeted mouse line from the KOMP depository that allowed testing the maternal effect of *Dot1l*, as illustrated in Fig. 1A. Flanking exon 2 by *loxP* sites in the floxed allele allows the conditional inactivation of the *Dot1l* gene. It was expected that deleting exon 2 using Cre-mediated recombination results in an out-of frame translated DOT1L protein, a null allele. We identified the floxed (Fig. 1B) and recombined (Fig. 1C) alleles using PCR assays (full gel image displayed in Fig. S1). First we confirmed that the *Dot1l*^–/–^ zygotic mutation of this mouse line results in embryonic lethality as reported earlier using independently derived *Dot1l* knockout lines^20,22^. We intercrossed *Dot1l*^+/–^ mice, and dissected the resulting embryos at 9.5, 10.5, and 11.5 days post coitum (dpc). Representative images are shown in Fig. 2A. We found that *Dot1l*^+/–^ embryos developed normally, but the *Dot1l*^–/–^ embryos were variably retarded at 9.5 and 10.5 dpc, and were dead and severely retarded at 11.5 dpc (Fig. 2B,C). We did not find any surviving *Dot1l*^–/–^ pups at birth. This result confirmed the embryonic lethality of the zygotic mutation in the *Dot1l* mouse line, and verified the suitability of the current mouse line for testing the maternal effect.

### *Dot1l* maternal mutant mice display normal development and fertility

**Figure 3:**
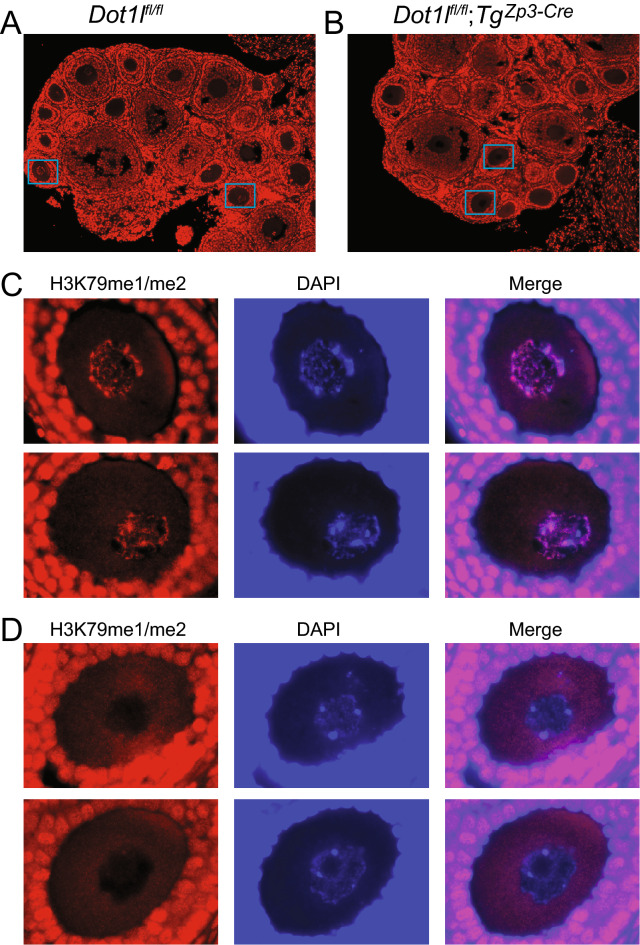
Maternal knockout of *Dot1l* eliminates H3K79me1/2 from *Dot1l*^*−/−*^ oocytes. (**A**) Representative picture of a normal 21-dpp ovary from a *Dot1l*^fl/fl^ mouse immunostained with an H3K79me1/me2 antibody (red). (**B**) Representative ovary of a 21-dpp *Dot1l*^fl/fl^*;* *Tg*^*Zp3-Cre*^ female mouse, which carries *Dot1l*^*−/−*^ oocytes. (**C**) Two selected oocytes from the *Dot1l*^fl/fl^ ovary above (marked by rectangles) are shown stained with an H3K79me1/me2 antibody (red) and counterstained with DNA marker DAPI (blue). (**D**) Two selected *Dot1l*^*−/−*^ oocytes from the *Dot1l*^fl/fl^*;* *Tg*^*Zp3-Cre*^ ovary above are shown stained with an H3K79me1/me2 antibody (red) and counterstained with DNA marker DAPI (blue). The diameter of these oocytes is 66–67 μm. Note the complete absence of H3K79me2 from the *Dot1l*^*−/*−^ oocyte nuclei. Granulosa cells are not affected by the oocyte-specific Cre-deletion.

We generated female *Dot1l*^fl/fl^*; Tg*^*Zp*3-*Cre*^ mice by crossing with the *Zp3-Cre* transgenic mice^46^. The mouse *Zona pellucida 3 (Zp3)* gene promoter drives the expression of *Cre* recombinase in these females, and Cre-excision is specifically targeted to growing oocytes. Zp3-Cre-mediated deletion of *Dnmt3a* and *Dnmt3b* occurred in growing oocytes between 3 and 10 days post partum (dpp)^35^. We previously used this strategy to generate *Ehmt2*^*mat–/*+^ and *Setdb1*^*mat–/*+^ maternal mutant zygotes^47^. To demonstrate that Cre-recombination inactivated the *Dot1l* gene in the oocytes we carried out an immunostaining experiment of *Dot1l*^fl/fl^; *Tg*^*Zp3-Cre*^ and control *Dot1l*^fl/fl^ ovaries at 21 dpp (Fig. 3A,B). We counted the oocytes with discernable DAPI DNA staining in the ovary sections of two females in each genotype. Among these, 15/15 *Dot1l*^fl/fl^ oocytes were positive for H3K79me1/2 staining, and 22/22 *Dot1l*^−/−^ oocytes were negative (Fig. 3C,D). This confirms that the Cre-recombination in growing oocytes has eliminated the DOT1L-dependent H3K79me1/2 staining completely by 21 dpp.

**Figure 4:**
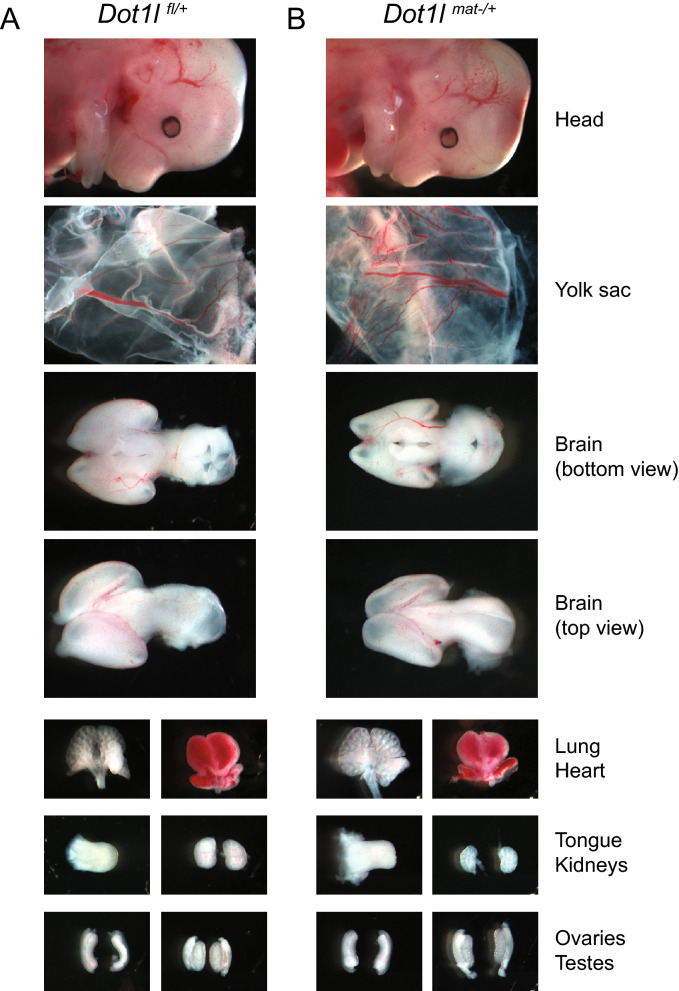
*Dot1l*^*mat−/*+^ maternal mutant embryos develop normally. (**A**) Representative pictures depict the head and major organs of a *Dot1l*^*fl/+*^ embryo at 13.5 dpc. (**B**) Representative pictures of a *Dot1l*^*mat−/*+^ embryo are shown at 13.5 dpc.

To test for maternal effect of phenotype, we crossed *Dot1l*^fl/fl^*; Tg*^*Zp3*-*Cre*^ female mice with JF1/Ms wild type males, and collected embryos at 13.5 dpc. The *Dot1l*^*mat–/*+^ embryos showed normal growth as compared with *Dot1l*^+/–^ embryos from the control cross using *Dot1L*^*fl/*+^*; Tg*^*Zp3*-*Cre*^ mothers (Table 1) and with the control *Dot1l*^*fl/+*^ embryos from *Dot1L*^*fl/fl*^ mothers. The heads and the major organs from representative 13.5 dpc *Dot1l*^*mat–/*+^ embryos and control *Dot1l*^*fl/+*^ embryos are shown in Fig. 4. Three *Dot1l*^*fl/*+^*; Tg*^*Zp3*-*Cre*^ mothers gave birth to a total of 21 *Dot1l*^*mat–/*+^ live pups. All of the *Dot1l*^*mat–/*+^ pups were grossly normal in growth and behavior (Table S1).

**Table 1:** Maternal deletion of *Dot1l* has no lethal effect in the F1 embryo.

Collection time	9.5 dpc		13.5 dpc	Birth to adult	
Mother	*Dot1l*^fl/fl^*; Tg*^*Zp3*-*Cre*^	*Dot1l*^fl/+^*; Tg*^*Zp3*-*Cre*^	*Dot1l*^fl/fl^*; Tg*^*Zp3*-*Cre*^	*Dot1l*^fl/fl^*; Tg*^*Zp3*-*Cre*^	*Dot1l*^fl/+^*; Tg*^*Zp3*-*Cre*^
Mother's egg	*Dot1l*^–/–^	*Dot1l*^–/+^	*Dot1l*^–/–^	*Dot1l*^–/–^	*Dot1l*^–/+^
Litters	1	2	1	3	1
Total pups	9	18	8	21	12
*Dot1l*^+/+^	0	13	0	0	ND
*Dot1l*^−/+^	9*	5	8*	21*	ND
Male	5	8	2	11	7
Female	4	10	6	10	5

The number of mothers and number of offspring (with genotypes) is tabulated from the different genetic crosses, as indicated by the mother’s and its oocyte’s genotype. The embryos were collected at 9.5 and 13.5 dpc, and the pups were followed from birth to adulthood.

*Dot1l^mat–/+^.

To detect potential subtle abnormalities caused by maternal DOT1L deficiency, we tested the fertility of the *Dot1l*^*mat–/*+^ animals. We intercrossed *Dot1l*^*mat−/*+^ females and *Dot1l*^*mat−/*+^ males. Four pairs of these breeders generated a total of 37 live pups from 7 litters, suggesting that the fertility and fecundity of *Dot1l*^*mat−/*+^ mice was not affected. 38% of these pups were *Dot1l*^+*/*+^, and 62% were *Dot1l*^+*/–*^, closely matching the Mendelian distribution of 1:2 (Table 2), as would also be expected from a *Dot1l*^+*/–*^ intercross (Fig. 2B). The *Dot1l*^+*/*+^ and *Dot1l*^+*/–*^pups appeared grossly normal.

**Table 2:** Maternal deletion of *Dot1l* has no grand-maternal effect.

Mating Pair	#1		#2		#3		#4		Total	
Number of litters	2		2		1		2		7	
Number of pups	16		10		5		6		37	
**Genotypes**										
*Dot1l*^+/+^	6	38%	5	50%	2	40%	1	17%	14	38%
*Dot1l*^+/−^	10	63%	5	50%	3	60%	5	83%	23	62%
*Dot1l*^−/−^	0	0%	0	0%	0	0%	0	0%	0	0%

The number of litters, pups, and their genotypes are tabulated from four mating pairs (#1–4) of *Dot1l*^*mat−/*+^ females and *Dot1l*^*mat−/*+^ males.

### *Dot1l* maternal mutation does not enhance the severity of the zygotic deletion

**Figure 5:**
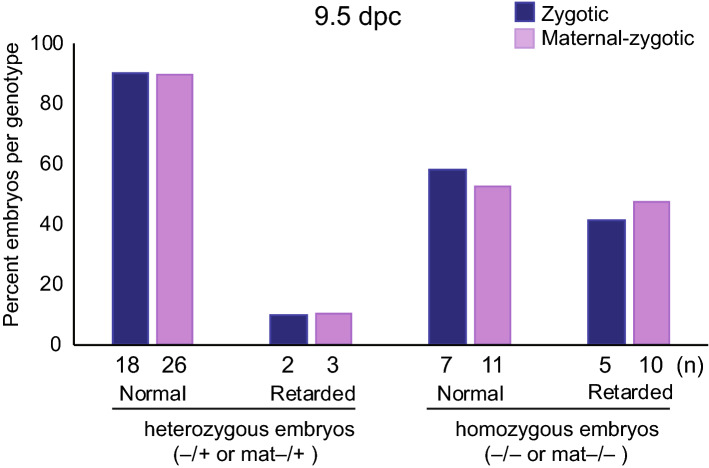
*Dot1l* maternal mutation does not enhance the severity of the zygotic deletion. *Dot1l*^fl/fl^*; Tg*^*Zp*3-*Cre*^ female mice were crossed with *Dot1l*^−/+^ male mice to generate offspring with two genotypes, *Dot1l*^mat−/−^ or *Dot1l* ^mat−/+^. As comparison, embryos with *Dot1l*^−/−^ and *Dot1l*^−/+^ were generated from the *Dot1l*^−/+^ intercross. The two crosses resulted in retarded embryos with similar frequencies at 9.5-dpc. The fraction of retarded embryos was similar between the *Dot1l*^mat−/−^ and *Dot1l*^−/−^ genotypes and between the *Dot1l*^mat−/+^ and *Dot1l*^−/+^ genotypes.

To test the possibility that maternally deposited DOT1L is only required in the absence of embryo-produced DOT1L, we combined the maternal mutation with the zygotic mutation. We crossed*Dot1l* ^fl/fl^*; Tg*^*Zp3*-*Cre*+^ females with *Dot1*^+*/–*^ males to generate *Dot1l*^mat–/–^ and *Dot1l*^mat–/+^ embryos, which we collected at 9.5 dpc. Five pregnant mothers resulted in a total of 40 embryos. We found that the frequency of retarded *Dot1l*^mat−/−^ embryos was similar to the frequency of *Dot1l*^–/–^ embryos generated from the *Dot1l*^–/+^ intercros (Fig. 5). The frequency of retarded *Dot1l*^mat–/+^ and *Dot1l*^–/+^ embryos was also similar in the two crosses. These results revealed that there is no extra harm from maternal DOT1L deletion in addition to the zygotic effect.

### *Dot1L* maternal deletion does not affect the parental allele-specific expression of imprinted genes at 9.5 dpc

**Figure 6:**
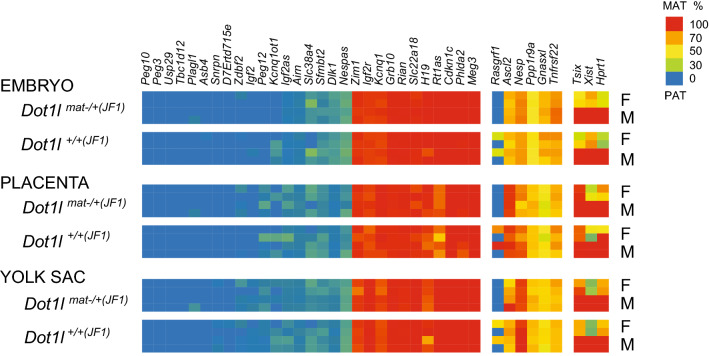
Parental allele-specific expression of the imprinted gene network is undisturbed in the *Dot1l* maternal mutant embryos. Parental allele-specific transcription was determined for 38 transcripts (marked at the top) in the 9.5 dpc embryo, placenta, and yolk sac using Sequenom allelotyping assays. *Dot1l*^*mat−/*+*(JF1)*^ embryos were obtained by crossing *Dot1l*^fl/fl^*; Tg*^*Zp*3-*Cre*^ female mice with JF1/Ms (JF1) wild type males. Control *Dot1l*^+*/*+*(JF1)*^ embryos were collected from *Dot1l*^fl/+^; *Tg*^*Zp*3-*Cre*^ females crossed with JF1/Ms wild type males. Allelotyping results from duplicate female (F) and male (M) samples are shown from the embryo, placenta, and yolk sac. The color scale to the right shows the percent of each transcript from the maternal allele in the total (maternal + paternal) expression. There was no change in the allelic expression between experimental and control groups.

We found earlier a reciprocal marking of the active, and inactive alleles of imprinted genes by the histone marks H3K79me2, and H3K9me3^31^. To test whether maternally deposited DOT1L was important for establishing, or maintaining these marks, we measured the allele-specific transcript levels of a large set of imprinted genes in the *Dot1l*^mat-/-^ embryos. We crossed *Dot1l*^fl/fl^*; Tg*^*Zp*3-*Cre*^ (C57BL/6J) females with JF1/Ms wild type males, and collected *Dot1l*^mat–(B6)/+(JF1)^ embryo, placenta, and yolk sac samples at 9.5dpc. We also set up the control cross of *Dot1l*^fl/+^*; Tg*^*Zp*3-*Cre*^^+^ (C57BL/6J) females with JF1/Ms wild type males and collected *Dot1l*^+(B6)/+(JF1)^ samples. We chose this control to closely match the genetic setup of the maternal mutant embryos including the Cre-recombination events that take place there. Whereas DOT1L was completely inactivated in the growing oocyte (Fig. 3) that gave rise to the maternal mutant zygote and embryo, it was expressed from one allele of *Dot1l* in the control oocytes. Haploinsufficiency does not result in a developmental phenotype (Fig. 2). We isolated RNA and carried out multiplex Sequenom allelotyping assays for 38 genes, including imprinted, and X chromosome-linked genes^31,48^. We found no difference in imprinted gene expression between *Dot1l*^mat−/+(JF1)^ embryos and control wild type *Dot1l*^+/+(JF1)^ embryos. There was also no deviation from the expected parental allelic biases^49,50^. None of these genes showed altered allele-specific transcription (Fig. 6). This result indicates that maternally deposited DOT1L is not essential for imprint establishment in the growing oocyte or protection of maternal imprints in the zygote.

In summary, maternal DOT1L deposition in growing oocytes is not required for normal development, and fertility of the offspring, or for imprinted gene expression.

## Discussion

We report that DOT1L and H3K79 methylation in the egg are dispensable for the first steps of mouse development. This conclusion is based on the findings that the maternal mutant (heterozygous) *Dot1l*^*mat–/*+^ offspring derived from mutant oocytes showed normal growth and fertility, even though no *Dot1l*^*–/–*^ homozygous null mutant mouse embryo survived beyond 10.5 dpc (Table 1)^22^. In addition, the lethality phenotype of the maternal-zygotic (homozygous) *Dot1l*^*mat–/–*^ mutant embryos was not more severe than that of the zygotic homozygous *Dot1l*^*–/–*^ mutant embryos.

The maternal mutation was carried out using the Zp3-Cre transgene^46^, which is known to effectively inactivate the floxed target in growing oocytes^14,35^. Cre-excision of the floxed *Dot1l* alleles in the growing oocytes is expected to have eliminated DOT1L protein and H3K79 methylation from oocytes at subsequent stages, including the germinal vesicle phase and metaphase I oocytes, in the zygote, and the 1-to 4 cell embryo. In the light of our findings, we conclude that the broad presence of H3K79me2 in the oocyte and in the zygote’s maternal pronucleus is dispensable for fertility. The localization of H3K79me3 at the pericentric heterochromatin of germinal vesicle oocytes and metaphase I oocytes is similarly dispensable. Reassembly of H3K79me2 to the chromosomes at the M-phase during preimplantation is not needed for embryo development. DOT1L and H3K79me2 are not required for the newly forming chromocenters at the four-cell stage. Elimination of H3K79me2 shortly after fertilization, and removal of DOT1L from the nucleus in the process of eliminating chromocenters at the 2-cell stage may be essential processes due to the presence of DOT1L during these stages. We cannot exclude the need for these processes in the light of the maternal knockout experiment, which renders them redundant by eliminating DOT1L from the oocyte. Removal of DOT1L from the 2-cell nucleus may, indeed, be necessary to reduce the magnitude of DSB repair and/or enhance totipotency of the 2-cell stage embryo.

Genomic imprinting is mediated by epigenetic modifications that differ on the two parental chromosomes and allow the gene to be expressed from only one parental allele. The methylation imprinting marks at the imprinted DMRs are deposited in the growing oocytes and are maintained in the zygote and later in the maternally inherited chromosome during somatic cell divisions. We deleted *Dot1l* specifically in growing oocytes to test if DOT1L is needed for maternal imprinting establishment and imprinting maintenance after fertilization. We found that the parental allele-specific transcription was not disturbed at a large set of imprinted genes examined, indicating the DOT1L is not essential for maternal imprint establishment nor imprint maintenance in the zygote.

To the best of our knowledge, this is the first report to show that maternal deposition of any histone methyltransferase is dispensable for mammalian development. The single cell organism, *S. cerevisiae* does not require *Dot1* in the vegetative cells^19^, but it does require DOT1 during the meiotic cell cycle, in a surveillance mechanism called the "pachytene checkpoint" to ensure proper chromosome segregation by preventing meiotic progression when recombination and chromosome synapsis are defective^25,51^. To test the role of DOT1L in female meiosis, Wang injected anti-*Dot1L* siRNA into mouse GV oocytes and found that the metaphase of meiosis was blocked^52^. They interpreted this finding to mean that the DOT1L mediated H3K79 methylation is essential to meiosis progression in mouse oocytes. Because we deleted *Dot1l* at an earlier stage and found no reduction in fertility, we think that the difference in the finding of Wang may be due to the different mouse strains or the in vitro approach applied. The first possibility cannot easily be tested, because those authors used the ICR outbred mouse, which is not suitable for genetic experiments, as it lacks the genetic homogeneity, which can be only attained by inbreeding. Our results cannot exclude the role of DOT1L in the early phase of female meiosis. Female germ cells enter meiosis at 13.5 dpc and stay in meiotic arrest in the pachytene stage until after birth. These stages were not affected in the Zp3-Cre deleted *Dot1l* mutant female germ line, which affects oocytes only after birth. DOT1L may also be required in the male germ line. H3K79me patterns, combined with the cytological analysis of the H3.3, gamma-H2AX, macroH2A and H2A.Z histone variants, are consistent with a differential role for these epigenetic marks in male mouse meiotic prophase I. H3K79me2 may be related to transcriptional reactivation on autosomes during pachynema, whereas H3K79me3 may contribute to the maintenance of repressive chromatin at centromeric regions and the sex body^53^. H3K79 methylation is specifically detected in the elongating spermatids in the fruit fly, rat, mouse and human species, preceding the histone-protamine exchange, suggesting that DOT1L may be an essential factor in male germ cells of higher order organisms. Indeed, *Drosophila gpp* mutant males exhibited reduced fertility^54,55^. Further genetic analysis is needed to reveal whether DOT1L is essential for histone-protamine exchange in mammalian spermiogenesis and in meiosis in the female and male germ lines.

## Material and methods

### Mouse breeding and genotyping

All animal experiments were performed according to the National Institutes of Health Guide for the Care and Use of Laboratory animals, with Institutional Care and Use Committee-approved protocols at Van Andel Institute (VAI).

Knockout First mouse line *Dot1l*^tm1a(KOMP)Wtsi^ (CSD29070 ) was obtained from KOMP Repository (https://www.komp.org/geneinfo.php?geneid=54455)^56^. This mouse was crossed with *ROSA26-FLPe* mouse B6N.129S4-Gt(ROSA)^26Sortm1(FLP1)Dym^/J (The Jackson Laboratory JAX# 016,226) to remove the LacZ and neo cassettes, resulting in the *Dot1l*^fl^ conditional knockout allele (Fig. 1). This line was maintained in a *Dot1l*^fl/fl^ homozygous breeding scheme.

Female *Dot1l*^fl/fl^ homozygous mice were crossed with a *Zp3-Cre* transgenic homozygous male purchased from The Jackson Laboratory (JAX#003651) to generate *Dot1l*^fl/+^*; Tg*^*Zp3*-*Cre*^ male, then this male further crossed with *Dot1l*^fl/fl^ females to generate *Dot1l*^fl/fl^*; Tg*^*Zp3*-*Cre*^ mice. Male mice were used as breeders to keep the line. Female *Dot1l*^fl/fl^*; Tg*^*Zp3*-*Cre*^ mice were used as experimental mothers for generating maternal mutant offspring. The second exon of the *Dot1l* gene was excised in their growing oocytes prior to the completion of the first meiotic division by the *Cre* recombinase driven by the mouse *Zona pellucida 3 (Zp3)* gene promoter. *Dot1l*^*fl/*+^; *Tg*^*Zp3-Cre*^ or *Dot1l*^*fl/fl*^ mice were used as control mothers. All *Dot1l* mutant mice were maintained in a C57BL/6J genetic background.

To generate heterozygous *Dot1l*^*–/*+^ mice we crossed *Dot1l*^fl/+^*; Tg*^*Zp3*-*Cre*^ female mice with C57BL6/J wild type males. *Dot1l*^*–/*+^ mice were gossly normal and fertile as expected.

The three *Dot1l* genotyping primers (Fig. 1) were: 5′-GTTTGTGGCTGTGCTGGACACA-3′; 5′-AAGGAAACAGAAGACGCAGCACTCC-3′; 5′-ATGCTTCAGAAAAAAGGCTGCAGAT-3′, are shown in Fig. 1. The *Cre* genotyping primers: 5′-TGCTGTTTCACTGGTTGTGCGGCG-3′ and 5′-TGCCTTCTCTACACCTGCGGTGCT-3′ resulted a 303 bp PCR product. The embryo sex was determined by *Sry* genotyping using: 5′-ATGGAGGGCCATGTCAAGCG-3′; 5′-TGCCACTCCTCTGTGACACTTTAG-3′ oligos, resulting a 275 bp PCR product.

### Embryo tissue collection

Female *Dot1l*^fl/fl^;* Tg*^*Zp*3*Cre*^ mice were crossed with males of different genotypes, as specified in the text. The embryo, placenta and yolk sac samples were collected at different developmental stages. Embryos were examined under a Zeiss Discovery 8 dissecting microscope and pictures were taken with ZEISS AxioCam ICc1 camera. We used 10 × magnification for the embryo and 20 × magnification for the organs. The end of embryo tail was used for genotyping by PCR using the oligonucleotide primers described above.

### Immunostaining of ovaries

Ovaries with adjacent tissue were taken out from three weeks old female mice and fixed in 4% paraformaldehyde at 4 °C for overnight, then dehydrated in a graded series of ethanol, and embedded in paraffin. Samples were sectioned at 5-μm thickness and stained with hematoxylin and eosin for histology check. For immunohistochemical analysis, 5-μm sections were deparaffinized and rehydrated in a graded series of ethanol to water, then pretreated by incubating with 1 mg/mL hyaluronidase (Sigma H4272) in PBS for 20 min at room temperature. Antigen retrieval was performed by incubating sections in citrate buffer (10 mM citric acid with 0.05% Tween20 at pH 6.0) heated to 95–100 °C for 40 min. Citrate buffer and samples were then transferred to room temperature and allowed to cool for an additional 60 min. After antigen retrieval, samples were permeabilized with 0.2% TritonX-100 in PBS for 20 min, then blocked with 10% normal goat serum and 1% BSA in PBS for 1 h. Primary antibody (custom preparation of rabbit polyclonal H3K79me1/me2 by Open Biosystems, now Themo Fisher)^57^ was then applied to sample and incubated at 4 °C for overnight. After washing with PBS, samples were incubated with Alexa 568-conjugated goat anti-rabbit secondary antibody (Invitrogen A11011) for 1 h at room temperature. After counterstaining with DAPI (0.5 μg/ml) for 10 min, samples were mounted with Prolong Gold antifade reagent (Invitrogen P36935) and sealed with coverslip. Fluorescence images were captured for ovaries at 100X magnification and for oocytes at 1000X magnification.

### Allele-specific RNA expression analysis

Total RNA from each tissue was extracted with RNA Bee (Tel Test), and further cleaned by DNA-free^TM^ DNA Removal Kit (Ambion) to remove any trace of DNA. The RNA concentration was measured using a Nanodrop spectrophotometer, and 400 ng of total RNA was reverse-transcribed in a 10 μL reaction volume using the SuperScript III random primer synthesis kit (Invitrogen) with random hexamers according to manufacture instruction.

To measure the contribution of each parental allele to total transcript levels we used a multiplex Sequenom (now Agena Bioscience) allelotyping assay as we described earlier^31,48^. The unextended extend primer (UEP) and PCR primer sequences are listed in Table S2. This method uses mass spectrometry quantification of the extended UEP primers based on the differences in molecular mass between alleles. Single nucleotide polymorphisms (SNPs) for the imprinted region were obtained by the DNA sequences of inbred C57BL6/J and JF1/Ms.

## Supplementary information

Supplementary Information 1.

Supplementary Information 2.

## References

[1] Eckersley-Maslin MA, Alda-Catalinas C, Reik W (2018). Dynamics of the epigenetic landscape during the maternal-to-zygotic transition. Nat. Rev. Mol. Cell Biol..

[2] Messerschmidt DM, Knowles BB, Solter D (2014). DNA methylation dynamics during epigenetic reprogramming in the germline and preimplantation embryos. Genes Dev..

[3] Zeng Y, Chen T (2019). DNA methylation reprogramming during mammalian development. Genes.

[4] Condic ML (2016). The role of maternal-effect genes in mammalian development: are mammalian embryos really an exception?. Stem Cell Rev. Rep..

[5] AuYeung WK (2019). Histone H3K9 methyltransferase g9a in oocytes is essential for preimplantation development but dispensable for CG methylation protection. Cell Rep..

[6] Brici D (2017). Setd1b, encoding a histone 3 lysine 4 methyltransferase, is a maternal effect gene required for the oogenic gene expression program. Development.

[7] Zylicz JJ (2018). G9a regulates temporal preimplantation developmental program and lineage segregation in blastocyst. eLife.

[8] Ancelin K (2016). Maternal LSD1/KDM1A is an essential regulator of chromatin and transcription landscapes during zygotic genome activation. eLife.

[9] Ciccone DN (2009). KDM1B is a histone H3K4 demethylase required to establish maternal genomic imprints. Nature.

[10] Sankar A (2017). Maternal expression of the histone demethylase Kdm4a is crucial for pre-implantation development. Development.

[11] Sankar A (2020). KDM4A regulates the maternal-to-zygotic transition by protecting broad H3K4me3 domains from H3K9me3 invasion in oocytes. Nat. Cell Biol..

[12] Wasson JA (2016). Maternally provided LSD1/KDM1A enables the maternal-to-zygotic transition and prevents defects that manifest postnatally. eLife.

[13] Eymery A, Liu Z, Ozonov EA, Stadler MB, Peters AH (2016). The methyltransferase Setdb1 is essential for meiosis and mitosis in mouse oocytes and early embryos. Development.

[14] Kim J (2016). Maternal Setdb1 is required for meiotic progression and preimplantation development in mouse. PLoS Genet.

[15] Singer MS (1998). Identification of high-copy disruptors of telomeric silencing in *Saccharomyces cerevisiae*. Genetics.

[16] Lacoste N, Utley RT, Hunter JM, Poirier GG, Cote J (2002). Disruptor of telomeric silencing-1 is a chromatin-specific histone H3 methyltransferase. J. Biol. Chem..

[17] Ng HH (2002). Lysine methylation within the globular domain of histone H3 by Dot1 is important for telomeric silencing and Sir protein association. Genes Dev..

[18] Takahashi YH (2011). Dot1 and histone H3K79 methylation in natural telomeric and HM silencing. Mol. Cell.

[19] Van Leeuwen F, Gafken PR, Gottschling DE (2002). Dot1p modulates silencing in yeast by methylation of the nucleosome core. Cell.

[20] Feng Y (2010). Early mammalian erythropoiesis requires the Dot1L methyltransferase. Blood.

[21] Shanower GA (2005). Characterization of the grappa gene, the *Drosophila* histone H3 lysine 79 methyltransferase. Genetics.

[22] Jones B (2008). The histone H3K79 methyltransferase Dot1L is essential for mammalian development and heterochromatin structure. PLoS Genet..

[23] Tatum D, Li S (2011). Evidence that the histone methyltransferase Dot1 mediates global genomic repair by methylating histone H3 on lysine 79. J. Biol. Chem..

[24] Franz H (2019). DOT1L promotes progenitor proliferation and primes neuronal layer identity in the developing cerebral cortex. Nucleic Acids Res..

[25] San-Segundo PA, Roeder GS (2000). Role for the silencing protein Dot1 in meiotic checkpoint control. Mol. Biol. Cell.

[26] Steger DJ (2008). DOT1L/KMT4 recruitment and H3K79 methylation are ubiquitously coupled with gene transcription in mammalian cells. Mol. Cell Biol..

[27] Godfrey L (2019). DOT1L inhibition reveals a distinct subset of enhancers dependent on H3K79 methylation. Nat. Commun..

[28] Ooga M (2008). Changes in H3K79 methylation during preimplantation development in mice. Biol. Reprod..

[29] Abe M, Tsai SY, Jin SG, Pfeifer GP, Szabo PE (2011). Sex-specific dynamics of global chromatin changes in fetal mouse germ cells. PLoS ONE.

[30] Singh P (2011). Chromosome-wide analysis of parental allele-specific chromatin and DNA methylation. Mol. Cell Biol..

[31] Singh P (2010). Allele-specific H3K79 Di- versus trimethylation distinguishes opposite parental alleles at imprinted regions. Mol. Cell Biol..

[32] Lucifero D, Mertineit C, Clarke HJ, Bestor TH, Trasler JM (2002). Methylation dynamics of imprinted genes in mouse germ cells. Genomics.

[33] Smallwood SA (2011). Dynamic CpG island methylation landscape in oocytes and preimplantation embryos. Nat. Genet..

[34] Bourc'his D, Xu GL, Lin CS, Bollman B, Bestor TH (2001). Dnmt3L and the establishment of maternal genomic imprints. Science.

[35] Kaneda M (2004). Essential role for de novo DNA methyltransferase Dnmt3a in paternal and maternal imprinting. Nature.

[36] Li X (2008). A maternal-zygotic effect gene, Zfp57, maintains both maternal and paternal imprints. Dev. Cell.

[37] Messerschmidt DM (2012). Trim28 is required for epigenetic stability during mouse oocyte to embryo transition. Science.

[38] Nakamura T (2007). PGC7/Stella protects against DNA demethylation in early embryogenesis. Nat. Cell Biol..

[39] Esse R, Gushchanskaia ES, Lord A, Grishok A (2019). DOT1L complex suppresses transcription from enhancer elements and ectopic RNAi in Caenorhabditis elegans. RNA.

[40] Cornelis FMF (2019). Increased susceptibility to develop spontaneous and post-traumatic osteoarthritis in Dot1l-deficient mice. Osteoarthr. Cartil..

[41] Jo SY, Domowicz MS, Henry JG, Schwartz NB (2020). The Role of Dot1l in Prenatal and Postnatal Murine Chondrocytes and Trabecular Bone. JBMR plus.

[42] Jo SY, Granowicz EM, Maillard I, Thomas D, Hess JL (2011). Requirement for Dot1l in murine postnatal hematopoiesis and leukemogenesis by MLL translocation. Blood.

[43] Monteagudo S (2017). DOT1L safeguards cartilage homeostasis and protects against osteoarthritis. Nat. Commun..

[44] Ooga M, Suzuki MG, Aoki F (2013). Involvement of DOT1L in the remodeling of heterochromatin configuration during early preimplantation development in mice. Biol. Reprod..

[45] Onder TT (2012). Chromatin-modifying enzymes as modulators of reprogramming. Nature.

[46] de Vries WN (2000). Expression of Cre recombinase in mouse oocytes: a means to study maternal effect genes. Genesis.

[47] Zeng TB, Han L, Pierce N, Pfeifer GP, Szabo PE (2019). EHMT2 and SETDB1 protect the maternal pronucleus from 5mC oxidation. Proc. Natl. Acad. Sci. USA.

[48] Iqbal K (2015). Deleterious effects of endocrine disruptors are corrected in the mammalian germline by epigenome reprogramming. Genome Biol..

[49] Andergassen D (2017). Mapping the mouse Allelome reveals tissue-specific regulation of allelic expression. eLife.

[50] Kang ER (2011). Effects of endocrine disruptors on imprinted gene expression in the mouse embryo. Epigenetics.

[51] Ontoso D, Acosta I, van Leeuwen F, Freire R, San-Segundo PA (2013). Dot1-dependent histone H3K79 methylation promotes activation of the Mek1 meiotic checkpoint effector kinase by regulating the Hop1 adaptor. PLoS Genet.

[52] Wang X (2014). Dot1L mediated histone H3 lysine79 methylation is essential to meiosis progression in mouse oocytes. Neuro Endocrinol. Lett..

[53] Ontoso D, Kauppi L, Keeney S, San-Segundo PA (2014). Dynamics of DOT1L localization and H3K79 methylation during meiotic prophase I in mouse spermatocytes. Chromosoma.

[54] Dottermusch-Heidel C (2014). H3K79 methylation: a new conserved mark that accompanies H4 hyperacetylation prior to histone-to-protamine transition in Drosophila and rat. Biol. Open.

[55] Dottermusch-Heidel C (2014). H3K79 methylation directly precedes the histone-to-protamine transition in mammalian spermatids and is sensitive to bacterial infections. Andrology.

[56] Skarnes WC (2011). A conditional knockout resource for the genome-wide study of mouse gene function. Nature.

[57] Singh P (2013). De novo DNA methylation in the male germ line occurs by default but is excluded at sites of H3K4 methylation. Cell Rep..

