# Supplementary material for: Maternal DOT1L is dispensable for mouse development
Source: Sci Rep. 2020 Nov 26;10:20636. doi: 10.1038/s41598-020-77545-6 (PMC7691351; doi:10.1038/s41598-020-77545-6)
Supplement: Supplementary file 1 — Supplementary Information 1. [file 41598_2020_77545_MOESM1_ESM.docx]

SUPPLEMENTARY FIGURE

**Figure S1. Full gel of the images displayed in Figure 1C and D.**

SUPPLEMENTARY TABLES

**Table S1. Daily monitoring of *Dot1l*^mat-/+^** **mice did not reveal the listed adverse effects.** Most importantly, all of the *Dot1l*^mat-/+^ mothers fed, and groomed the newborns until they were weaned, and they also carried pregnancies at normal intervals.

1. Slow, shallow, labored breathing

2. Hunched posture

3. Ruffled fur (for 3 days), failure to groom

4. Rapid weight loss (20-25% in one week)

5. Anorexia, slow steady weight loss

6. Hypo- or hyper-thermia

7. Elevated respiratory rate

8. Diarrhea or constipation (for 3 days)

9. Skin sores, infections, necrotic tissues and tumors

10. Lethargy (for 3 days)

11. Impaired mobility/lesions restricting access to water/food

12. Any persistent bleeding

13. Paralysis

14. CNS signs (seizures, spasticity, weakness)

15. Persistent self-induced trauma or severe repetitive behavior

16. Self-segregation from other animals

**Table S2. Sequenom allelotyping primers.**

| Sequence Name | Sequence |
| --- | --- |
| Cdkn1c chr7 150644367_UEP | GCT TAC AGT GTC CCC |
| Peg10 chr6 4707869_UEP | CCT GAA GCC ACC AGA |
| Kcnq1 chr7 150380204_UEP | GGA CAG AAG CGT GTG |
| lgf2as chr7 149853327_UEP | CAG AGC TCC TGC AAA T |
| Meg3 chr12 110783250_UEP | TGA CCC CTT CTC TTA CC |
| Rasgrf1 chr9 89886484_UEP | ACC TTC CTG CAT TCC TA |
| Slc38a4 chr15 96825404_UEP | GAC TCC AGG GCA GAT AC |
| *Igf2* chr7 149836880_UEP | TCA GTG GGA TCC CCT CTT |
| Gnasxl chr2 174124224_UEP | GCC ATT GAC AGA CCA TTC |
| Tnfrsf22 chr7 150823107_UEP | GCT TCT CCC CTC TAC TTG C |
| Peg12 chr7 69608449_UEP | GAA GGT CGT CCC TAG TTC T |
| Slc22a18 chr7 150682910_UEP | ACA TTT TCC AGA GGA AGC C |
| Rian chr12 110884294_UEP | AAC AAG CAT GTT TGA TGA T |
| Phlda2 chr7 150688294_UEP | TCT AAG CGC TTC TTC TTC CA |
| D7Ertd715e (a) chr7 67115005_UEP | TTT GGC ATG CAC TAT AAA AG |
| Sfmbt2 chr2 10512778_UEP | GAA TTT TCA CAA GTA TGG CG |
| Rt1as chr12 110831619_UEP | CTC TCG CGA TCA CCT CTT CTT |
| Mest chr6 30695854_UEP | CCA GCG TCT TCT GAA TTT CTT |
| Peg3(a) chr7 6660886_UEP | CCC GGG CAG GAA TCT TCC TTC |
| Nespas chr2 174108813_UEP | GCC GCA GGT CGT TTT ACC TTG |
| Ascl2 chr7 150152999_UEP | ATA TTT TCA GTA GAG TCC TAC A |
| Zim1 chr7 6629191_UEP | CCC ATC CAA GTG TCT ACA TTT AA |
| Ppp1r9a chr6 5111428_UEP | ACA TTA TCT GAA CTA GAA ACT TA |
| Kcnq1ot1 chr7 150427528_UEP | CCC CCT TTT TAA ATC CAT GAG TTG |
| Plagl1 chr10 12844715_UEP | AGG CTG TAG CGC TGC |
| Asb4(b) chr6 5373400_UEP | AAC CCC TGC ACA CGG C |
| Dlk1 chr2 110698537_UEP | CAG CCT CCT TGT TGA A |
| Grb10 chr11 11831012_UEP | GGT GGT TAC ACG GAG T |
| *Igf2*r chr17 12876894_UEP | AGG ACG GGC AAG AAA A |
| Usp29 chr7 6918737_UEP | TTC TGG CTT CGT AGT AC |
| Nesp chr2 174110831_UEP | GGG CGG GGC TGC CTG TT |
| Zdbf2 chr1 63360418_UEP | ACT ACC AGA ACC TTT CTC |
| Snrpn chr7 67127862_UEP | GAC ATT TGC TCA AGC TAG |
| *H19* chr7 149761651_UEP | GCA GCA TTG CCA AAG AGG |
| Airn(a) chr7 12948949_UEP | CCA CAA AGA CAG ACA GCT TC |
| Tbc1d12 chr19 38993732_UEP | ACC CAT AGG GTA AAA ATA TAT G |
| Cdkn1c chr7 150644367_for | ACG TTG GAT GCC CCA CAC ATT CAT CTT CAG |
| Cdkn1c chr7 150644367_rev | ACG TTG GAT GCA TTT CGA CTG TCT GGT CAC |
| Peg10 chr6 4707869_for | ACG TTG GAT GGA CTG AGA GGT GGT TAC AAC |
| Peg10 chr6 4707869_rev | ACG TTG GAT GAA GTG TGA CAC TTG GCA ACC |
| Kcnq1 chr7 150380204_for | ACG TTG GAT GCT TTT TAG GTT TGG GGC TGG |
| Kcnq1 chr7 150380204_rev | ACG TTG GAT GGG AAG ATC TAT GTC CGG AAG |
| lgf2as chr7 149853327_for | ACG TTG GAT GAT CTC TAG CAC AGG AGC ATC |
| lgf2as chr7 149853327_rev | ACG TTG GAT GGA CTA GAC TTC CTA GCC TTG |
| Meg3 chr12 110783250_for | ACG TTG GAT GTG TCC AAC TTT GAC CCC TTC |
| Meg3 chr12 110783250_rev | ACG TTG GAT GAT TCA CAC GGA GGA CAC TTG |
| Rasgrf1 chr9 89886484_for | ACG TTG GAT GCT TTC TCA ACA CCT TCC TGC |
| Rasgrf1 chr9 89886484_rev | ACG TTG GAT GTC GCA CTC ATG GGT TTT CTG |
| Slc38a4 chr15 96825404_for | ACG TTG GAT GAG AGT TCA CAC AGA CTC CAG |
| Slc38a4 chr15 96825404_rev | ACG TTG GAT GCC CCA GTA CAT TGA ACC GTC |
| *Igf2* chr7 149836880_for | ACG TTG GAT GGT CTC TTC CCT ACT GTC TTC |
| *Igf2* chr7 149836880_rev | ACG TTG GAT GAC AGG TGA CAT ATG GCG TTC |
| Gnasxl chr2 174124224_for | ACG TTG GAT GTC CGA GGC GCC ATT GAC AGA |
| Gnasxl chr2 174124224_rev | ACG TTG GAT GGC TGT CGC ATG CGA AGT TAG |
| Tnfrsf22 chr7 150823107_for | ACG TTG GAT GCC AAA CCT GGA ATT CAC CTC |
| Tnfrsf22 chr7 150823107_rev | ACG TTG GAT GTT TCC TGA TGT GCT GAG GAG |
| Peg12 chr7 69608449_for | ACG TTG GAT GTA TTA GCT GCC AGG GAC AAG |
| Peg12 chr7 69608449_rev | ACG TTG GAT GAC CCT GGC GAT TGT GAT GGA |
| Slc22a18 chr7 150682910_for | ACG TTG GAT GTG AGC ACC CAT TTT CCA GAG |
| Slc22a18 chr7 150682910_rev | ACG TTG GAT GAG TCC CAC AAC AGC AAA GAC |
| Rian chr12 110884294_for | ACG TTG GAT GTG TTA CAC CTC TTC TCA TCC |
| Rian chr12 110884294_rev | ACG TTG GAT GTG CCA GTC ATT CTG AGA AAC |
| Phlda2 chr7 150688294_for | ACG TTG GAT GTG GTT TTC CCG GAG AAG AGG |
| Phlda2 chr7 150688294_rev | ACG TTG GAT GAA GCG AAG CGA CAG CCT GTT |
| D7Ertd715e (a) chr7 67115005_for | ACG TTG GAT GAC CCA AAA TTT GGC ATG CAC |
| D7Ertd715e (a) chr7 67115005_rev | ACG TTG GAT GAT ACC AGG TAC TGT TAG GGC |
| Sfmbt2 chr2 10512778_for | ACG TTG GAT GTC TGT CAG AGG TGC CTT TTC |
| Sfmbt2 chr2 10512778_rev | ACG TTG GAT GAT GTC CCA GGC TTT GCT TAC |
| Rt1as chr12 110831619_for | ACG TTG GAT GAG ACA TTG ATC GCG ATC ACC |
| Rt1as chr12 110831619_rev | ACG TTG GAT GAT GTA GTT CCC TTC TGC TCC |
| Mest chr6 30695854_for | ACG TTG GAT GGA TGG ACA CAG AAG CAA GCG |
| Mest chr6 30695854_rev | ACG TTG GAT GGG TCA TCG ACA GTC TTT TAC |
| Peg3(a) chr7 6660886_for | ACG TTG GAT GCA TCT TCT CTG GCA GGA ATC |
| Peg3(a) chr7 6660886_rev | ACG TTG GAT GAC GTC ATA GAG CAA ATG ACC |
| Nespas chr2 174108813_for | ACG TTG GAT GAG TCC ATC GGC TCT TCC AAG |
| Nespas chr2 174108813_rev | ACG TTG GAT GTG GAT CCT GCT GTT GGA CTC |
| Ascl2 chr7 150152999_for | ACG TTG GAT GCC AAG CTT AAG GAT CCT TTG |
| Ascl2 chr7 150152999_rev | ACG TTG GAT GGG ACT TGG AAA TTT CTC AGG |
| Zim1 chr7 6629191_for | ACG TTG GAT GGC CAA TGA AGA CTG GGA ATG |
| Zim1 chr7 6629191_rev | ACG TTG GAT GCC ACT AAT GAG ACA TGG CCG |
| Ppp1r9a chr6 5111428_for | ACG TTG GAT GCT GTG TGG CCT CAC TTT GTA |
| Ppp1r9a chr6 5111428_rev | ACG TTG GAT GAA GCA GGC TCT GGG AGA AAG |
| Kcnq1ot1 chr7 150427528_for | ACG TTG GAT GTA TGT TCC AGA GTT GTT GGG |
| Kcnq1ot1 chr7 150427528_rev | ACG TTG GAT GGG ACA CAT GCA TGC ATC TGA |
| Plagl1 chr10 12844715_for | ACG TTG GAT GGC TAT TGT GCT CTG GAT CTC |
| Plagl1 chr10 12844715_rev | ACG TTG GAT GAA GCC CAG ACA GAA AGA AGG |
| Asb4(b) chr6 5373400_for | ACG TTG GAT GCA ACC AGG ATG AAG AGA CAC |
| Asb4(b) chr6 5373400_rev | ACG TTG GAT GTT CTC CAC GTA GAA GGC AAC |
| Dlk1 chr2 110698537_for | ACG TTG GAT GGC TGC TTA GAT CTC CTC ATC |
| Dlk1 chr2 110698537_rev | ACG TTG GAT GGC GGT CAA TAT CAT CTT CCC |
| Grb10 chr11 11831012_for | ACG TTG GAT GCC TTC TCA ACT TAG TCC AGG |
| Grb10 chr11 11831012_rev | ACG TTG GAT GGA CTT TGT AAT CTC TGC CTG |
| *Igf2*r chr17 12876894_for | ACG TTG GAT GTG ATG GAA GAG ATC CAG GTG |
| *Igf2*r chr17 12876894_rev | ACG TTG GAT GAG AGA GCT TCT GCT TTC ACC |
| Usp29 chr7 6918737_for | ACG TTG GAT GAA CTG GCC TCT TCT GGC TTC |
| Usp29 chr7 6918737_rev | ACG TTG GAT GGG TAC CCC TTT GAA GGT AAA |
| Nesp chr2 174110831_for | ACG TTG GAT GTG GCC TCC TGG TCT TGC AGC |
| Nesp chr2 174110831_rev | ACG TTG GAT GTA GAT CAG GAT CCT CGG GAC |
| Zdbf2 chr1 63360418_for | ACG TTG GAT GAT GAC ACC ACT ACC AGA ACC |
| Zdbf2 chr1 63360418_rev | ACG TTG GAT GAA CCA TGC AGG GAA CAA AGC |
| Snrpn chr7 67127862_for | ACG TTG GAT GCA CAG ATA TGA CAT TTG CTC |
| Snrpn chr7 67127862_rev | ACG TTG GAT GAG TGA ATG TCA GAA ATC AGG |
| *H19* chr7 149761651_for | ACG TTG GAT GTT GCC CTC AGA CGG AGA TG |
| *H19* chr7 149761651_rev | ACG TTG GAT GGC TTT GAG TCT CTC CGT ATG |
| Airn(a) chr7 12948949_for | ACG TTG GAT GAC CAG ACC ACA AAG ACA GAC |
| Airn(a) chr7 12948949_rev | ACG TTG GAT GTT GGA TAG TTT AGG GTC GGG |
| Tbc1d12 chr19 38993732_for | ACG TTG GAT GGA GGC TAG AAT GAT CAA CCC |
| Tbc1d12 chr19 38993732_rev | ACG TTG GAT GAA CTG CCT TAG GAG TAT GGG |
