# Supplementary figures and images for: Maternal DOT1L is dispensable for mouse development
Source: Sci Rep. 2020 Nov 26;10:20636. doi: 10.1038/s41598-020-77545-6 (PMC7691351; doi:10.1038/s41598-020-77545-6)

Liao Figure S1

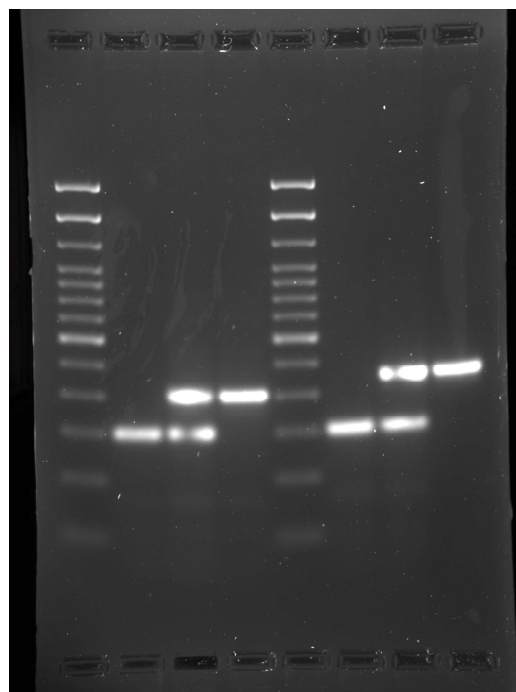

Supplement: Supplementary file 2 — Supplementary Information 2. [file 41598_2020_77545_MOESM2_ESM.pdf]
